# Bayesian spatio-temporal modelling and mapping of malaria and anaemia among children between 0 and 59 months in Nigeria

**DOI:** 10.1186/s12936-022-04319-y

**Published:** 2022-11-01

**Authors:** Jecinta U. Ibeji, Henry Mwambi, Abdul-Karim Iddrisu

**Affiliations:** 1 School of Mathematics, Statistics and Computer Science, University of KwaZulu Natal, Durban, South Africa; 2 School of Science, Mathematics and Statistics, University of Energy and Natural Resources, Sunyani, Ghana

**Keywords:** Spatio-temporal, Heterogeneity, Bayesian Hierarchical, Deviance Information Criteria, Risk Ratio and R-integrated nested Laplace approximation (INLA)

## Abstract

**Background/M&M:**

A vital aspect of disease management and policy making lies in the understanding of the universal distribution of diseases. Nevertheless, due to differences all-over host groups and space–time outbreak activities, data are subject to intricacies. Herein, Bayesian spatio-temporal models were proposed to model and map malaria and anaemia risk ratio in space and time as well as to ascertain risk factors related to these diseases and the most endemic states in Nigeria. Parameter estimation was performed by employing the R-integrated nested Laplace approximation (INLA) package and Deviance Information Criteria were applied to select the best model.

**Results:**

In malaria, model 7 which basically suggests that previous trend of an event cannot account for future trend i.e., Interaction with one random time effect (random walk) has the least deviance. On the other hand, model 6 assumes that previous event can be used to predict future event i.e., (Interaction with one random time effect (ar1)) gave the least deviance in anaemia.

**Discussion:**

For malaria and anaemia, models 7 and 6 were selected to model and map these diseases in Nigeria, because these models have the capacity to receive strength from adjacent states, in a manner that neighbouring states have the same risk. Changes in risk and clustering with a high record of these diseases among states in Nigeria was observed. However, despite these changes, the total risk of malaria and anaemia for 2010 and 2015 was unaffected.

**Conclusion:**

Notwithstanding the methods applied, this study will be valuable to the advancement of a spatio-temporal approach for analyzing malaria and anaemia risk in Nigeria.

## Background

Childhood malaria infection has been a major concern, especially in developing countries like Nigeria. The 2021 report from the World Health Organization (WHO) estimated that 241 million malaria cases with 627,000 deaths worldwide [1, 2]. The increase in malaria infection dropped from 81% in 2000 to 59% in 2015 and 56% in 2016 but went up again to 59% in 2020 due to Covid-19 pandemic. Globally, 96% of malaria cases is majorly from 29 countries with Nigeria (27%) topping the list of 6 countries that contribute almost 55% of malaria cases [1, 3]. Children between the age of 0 to 59 months are most vulnerable with an estimate of 213 million to 228 million malaria cases between 2019 and 2020 and a mortality rate of 534,000 to 602,000 in the respective years; 80% of all malaria deaths are among children under age 5 years [1, 4]. Like most other vector borne diseases, malaria is characterized by spatio-temporal variations or changes due to demographic, socio-economic and geographical factors. These covariates can help determine the spatio-temporal patterns of disease and recognize hotspots to aid efficient examination of disease, cost-effective allocation of resources and most importantly, effective disease control [5–10].

The spatio-temporal distribution of vector borne diseases is not only determined by environmental factors. Political and state borders are also major determinants because of their involvement in the spatial distribution and enactment of control and prevention programmes [10]. This can be explained by a spatio-temporal study in Northern Thailand, where there was a sharp difference in the malaria prevalence with Myanmar border [7]. Also, only environmental and biological factors cannot justify the differences in local diseases as claimed by Ra et al.[6].

Anaemia is another disease that has become a public health challenge in Nigeria, especially amongst under aged 5 years children. It is a condition that arises as a result of the reduction of hemoglobin in the blood [11, 12]. Globally, more than 273 million children under aged 5 years are affected by anaemia [12]. Sub-Sahara Africa is the most endemic region with about 53.8% childhood anaemia cases [12]. According to the WHO classification, anaemia is considered severe if its prevalence is 40% and above, moderate between 20% and 39.9%, and mild between 5% and 19.9% [12, 13]. Anaemia has become major public health due to its prevalence and effect on child’s health.

Pregnant women and children are most vulnerable to anaemia because of their high requirement of iron. Children between the age of 6–59 months are anaemic if their haemoglobin level is below 11 g/dl. The major causes of anaemia in children are parasitic infection, dietary iron deficiency and inherited disorders but in malaria endemic region, malaria disease is the major cause [14, 15].

The spread of malaria and anaemia in Nigeria has been a concern to researchers which has led to several studies such as [12, 16–20]. In [21], a quasi-experimental fixed-effect model was used to investigate the effect of malaria on haemoglobin concentration in children under 5 years old. They concluded that there is a strong negative effect on haemoglobin levels among Burkina Faso’s children from malaria infection. Furthermore, [22] stated that anaemia caused by *Plasmodium falciparum* is a result of excess removal of nonparasitized red blood cells together with the destruction of parasitized red cells immune leading to malfunctioning of the bone marrow. Also, the main cause of mortality and morbidity in children who live in Kenyan malaria hotspots is falciparum malaria [23]. Given these literatures and more, there is a need to monitor the progress of malaria and anaemia in the near future and use the available data to forecast cases in space and for the possible spread of these diseases.

The spatial pattern of diseases and exposures does not explain the temporal variation which is also important and interesting. Besag et al. [24], foremost introduced a spatial pattern which was extended by incorporating a linear time trend for interaction [25]. Knorr‐Held [26], included a non-parametric temporal trend which comprises the time changing effect of predictors. Including disease surveillance studies, spatio-temporal models are mostly used in several fields of science [27]. With the help of Bayesian hierarchical modelling framework, the implementation of these models is made possible. These models accommodate a composite and workable structure in space and time models, with spatio-temporal interaction as the paramount feature. Here, our work is extended from the methods used by Bernardinelli et al*.* [25], and Knorr‐Held [26] for spatio-temporal framework. By applying the multilevel model analysis [27], we independently investigate the spatio-temporal distribution of malaria and anaemia and their associated risk factors using data from the Nigeria malaria indicator survey (NMIS) for 2010 and 2015.

### Data description

**Fig. 1:**
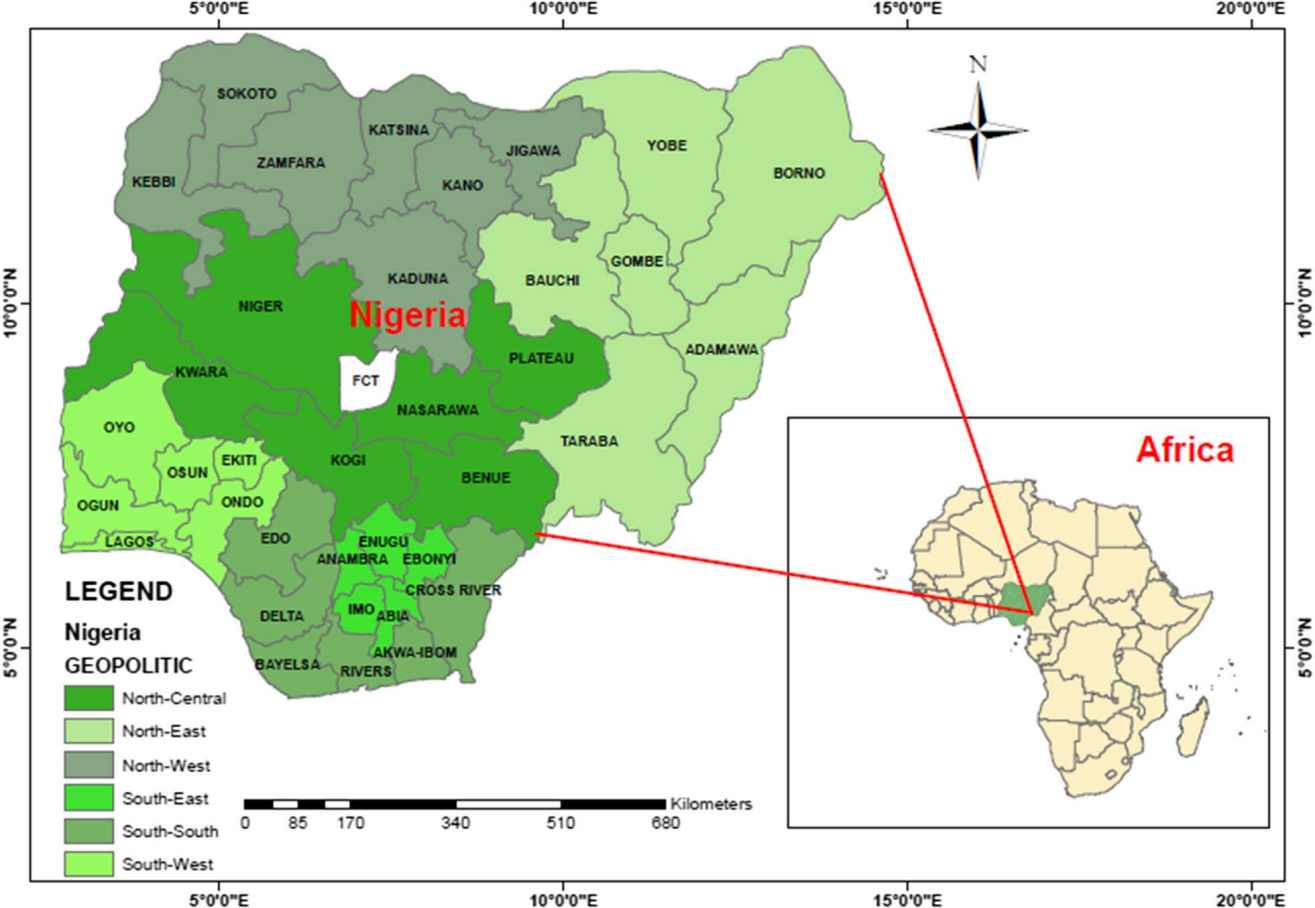
Location map of Nigeria showing the 6 geopolitical zones and their 37 states including the capital territory

**Fig. 2:**
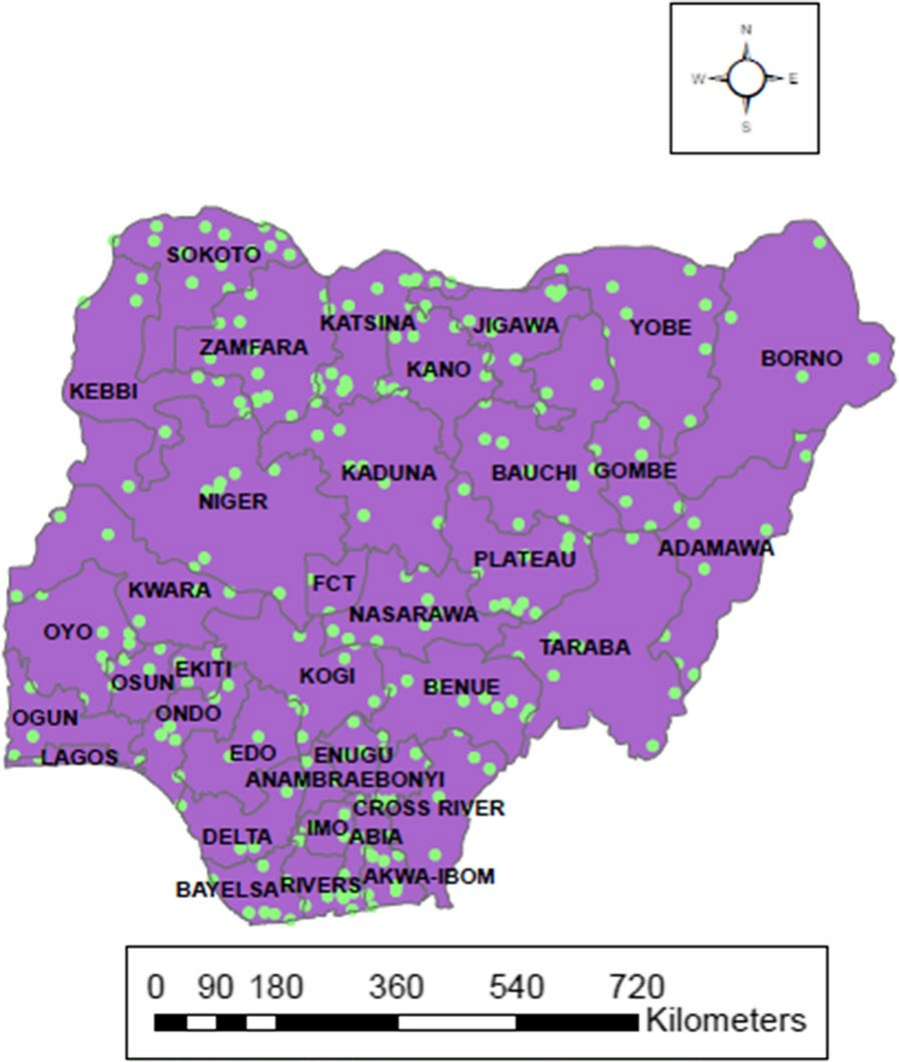
Map of Nigeria showing state rates based on sampling weights of under 5 years old malaria prevalence

**Fig. 3:**
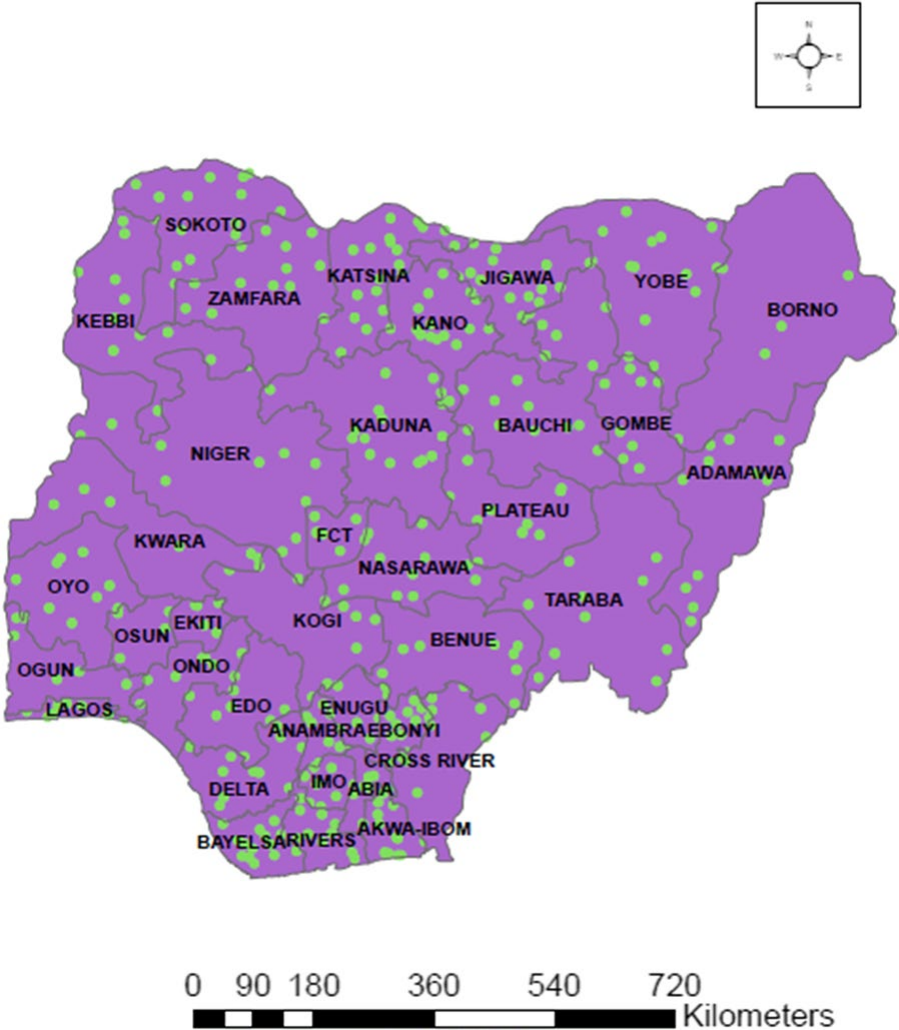
Map of Nigeria showing state rates based on sampling weights of under 5 years old anaemia prevalence

This work obtained data from the Nigeria Malaria Indicator Survey (NMIS) which was carried out by the National Malaria Elimination Program (NMEP), National Population Commission (NPopC) and the National Bureau of Statistics (NBS). The data captures the surveys of 2010 and 2015 which were the first and second malaria indicator surveys conducted in Nigeria. The 2015 survey was put into action just a year after the 2010 survey and a year after the development of the new national malaria strategic plan that covers 2014–2020 [28]. The 2 years were used because this survey is usually carried out every 5 years. To find out about the risk of malaria or anaemia disease, two-stage sampling was carried out. Clusters were selected from each urban/rural strata in the first stage and systematic sampling were done for selection of households in the second stage. The data has 12,623 children under age 5 years old in total. 11,172 and 11,072 children were tested for malaria and anaemia out of 12,623 respectively. Finally, the sample size of 9,533 was used for analysis after removing the missing values and this is 75.5% of the original data. Figure 1 shows the map of Nigeria comprising 6 geopolitical zones and their 37 states including the capital territory. It further reveals the location of Nigeria in Africa. While the maps in Figs. 2 and 3 indicate the prevalence of malaria and anaemia in each state.

In this study, the first dependent variable is the binary response from a child’s RDT outcome while the second dependent variable is the binary response to the anaemic status of a child. For both dependent variables, 1 represents the presence of malaria or anaemia infection and 0 represents no presence of malaria or anaemia infection. The independent variables are the type of place of residence, source of drinking water, type of toilet facility, has electricity, has radio, has television, main floor material, main wall material, main roof material, wealth index, child’s age in months, sex, mother’s highest educational level, state, and region. Table 1 contains the summary of all the variables used in this work.

**Table 1:** Exploratory data analysis

Variables	Category	Malaria		Anaemia	
		Positive (%)	Negative (%)	Positive (%)	Negative (%)
Type of place of residence	Urban	8.4	22.5	17.9	13
	Rural	36.9	32.2	50.5	18.6
Source of drinking water	Tap/Other water	17.5	22.3	26.6	13.2
	Well water	27.7	32.5	42	18.2
Wealth index	Poor	23.3	15.4	28.6	10
	Middle	10.8	10.9	15.5	6.2
	Rich	11.1	28.5	24.2	15.5
Region	North Central	8.6	10.1	11.2	7.5
	Northeast	7.8	10.7	11.6	7
	Northwest	13.9	10.8	19.1	5.1
	Southeast	3.7	7.4	7.3	3.8
	South South	6.7	8.9	12	3.8
	Southwest	4.4	7	7.3	4.3
Child's age in months	6–14 months	5.9	11.2	13.2	3.8
	15–23 months	5.8	8.9	11.2	3.5
	24–32 months	7.6	9.3	12	4.9
	33–41 months	8.4	9.2	11.4	6.2
	42–50 months	8.3	8.1	10.1	6.3
	51–59 months	9.3	8	17.4	6.8
Sex	Male	23.1	27.4	35.5	15
	Female	22.1	27.4	32.9	16.6
Electricity	No	30	22.4	38.6	13.9
	Yes	15.2	32.4	29.7	17.8
Television	No	31.7	24.8	41.6	14.7
	Yes	13.6	29.9	26.8	16.9
Radio	No	18	16.7	42.3	25.6
	Yes	27.2	38.1	22.5	9.2
Mother's educational level	No education	24.9	19.8	32.9	11.5
	Primary	9.2	10.2	13.5	6
	Secondary	9.6	9.9	18.9	10.8
	Higher	1.1	5.3	3.3	3.1
Main floor material	Earth/Other	25	21.4	33.3	13.1
	Cement/Ceramics	20.2	33.4	35.1	18.5
Main wall material	Wood/Other	28	22	36.2	13.6
	Cement/Bricks	17.2	32.8	32.2	18
Main roof material	Wood/Other	17.4	14.2	22.7	8.9
	Zinc/Metal	27.9	40.5	45.6	22.8
Type of toilet facility	Flush/Other toilet	22.7	25.4	32.8	15.7
	Pit toilet	22.5	29.4	35.5	16

## Methods

To begin with, a space–time model was proposed by Bernardinelli et al. [25] using Poisson distribution and the log risk ratio was defined as a linear function of time. Authors expressed the log risk ratio for area $i$; $i=1,\dots ,I$ for time $t$; $t=1,\dots ,T$ as

1 $$log\left({\theta }_{it}\right)={\eta }_{it}=\mu +{u}_{i}+{v}_{i}+\left(\beta +{\delta }_{i}\right)\times t.$$

Following Besag et al. [24] specification, $\phi ={u}_{i}+{v}_{i}$ are the spatial random effect (convolution model), where ${u}_{i}$’s are the structured variables and ${v}_{i}$’s are the unstructured variables. $\mu$ is the overall mean, $\beta$ is the universal linear time trend effect and ${\delta }_{i}$ is the random effect for interaction betwixt space and time. For the data to show the time trend, the parameters for the time trend were allocated unclear priors. In terms of the interaction random effect ${\delta }_{i}$, the independent and identically distributed (i.i.d) Gaussian prior was adopted, though alternative prior specification can be given. Based on spatial models, the priors for unstructured and structured spatial effects were described.

On the other hand, Knorr‐Held [26] reformed the earlier method by disabling the parametric limitations. The authors adopted a Binomial distribution for the number of cases in the country $i$ $\left(i=1,\dots ,I\right)$ at $t$ th time $\left(t=1,\dots ,T\right)$, while the log odds are expressed as:

2 $$log\left(\frac{{\pi }_{it}}{1-{\pi }_{it}}\right)={\eta }_{it}=\mu +{u}_{i}+{\upsilon }_{i}+{\gamma }_{t}+{\nu }_{t}+{\delta }_{it}$$

where ${\gamma }_{t}$, ${\nu }_{t}$ refers to the temporal random effects that take care of unnamed attributes of year $t$, and ${\delta }_{it}$; the interaction effects that take care of differences not described by the main effects. Intrinsic conditional autoregression (iCAR) and first order random walk structure were assigned to ${u}_{i}$ and ${\gamma }_{t}$, while independent Gaussian priors were assigned to ${\upsilon }_{i}$ and ${\nu }_{t}$. Based on the temporal effects interaction and spatial effects, the interaction ${\delta }_{it}$ was presumed to have four forms of prior inference.

Bernardinelli et al. [25] and Knorr‐Held [26] performed their parameter estimation under the fully Bayesian approach with the use of Markov chain Monte Carlo (MCMC) through Gibbs sampling techniques. Here, INLA approximation to fully Bayesian estimation was used. Therefore, the method used in this study is discussed as follows.

Let ${y}_{iky}$ be malaria or anaemia status of child $k$ in state $i$: $i=1,\dots ,37$ during year $y$: $y=\mathrm{1,2}$. The response outcome variable is a binary response, and it is defined as:

$$Y_{{iky}} = \left\{ {\begin{array}{*{20}c} {1,{\text{Anaemia}}} \\ {0,{\text{No}}\,{\text{anaemia}}} \\ \end{array} } \right.$$

where ${y}_{iky}$ is the binary response outcome and it follows a Bernoulli distribution as.

${y}_{iky}\sim Bernoulli\left({\theta }_{iky}\right)$, where ${\theta }_{iky}$ are unspecified probabilities associated to the outcome probabilities of the models. The logistic regression model is expressed as;

3 $$logit\left({\theta }_{iky}\right)={\beta }_{0}+{\eta }_{iky}$$

where ${\beta }_{0}$ is the model intercept and the linear predictor ${\eta }_{iky}={\mathrm{\rm X}}_{iky}^{^{\prime}}\beta$ with covariate vector $\mathrm{\rm X}={\left({x}_{iky1},\dots ,{x}_{ikyq}\right)}^{^{\prime}},$ $\beta =\left({\beta }_{1},\dots ,{\beta }_{q}\right)$ is the vector regression coefficient. We employed the combined formulation of the structured additive regression to permit flexibility where the classical predictor can be expanded to a better flexible additive predictor. Therefore, the structured additive predictor is expanded to spatio-temporal modelling as

4 $${\eta }_{iky}={\mathrm{\rm X}}_{iky}^{^{\prime}}\beta +{f}_{spat}\left({s}_{i}\right)+{f}_{year}\left(y\right)+{f}_{iy}\left({s}_{i},y\right)$$

where ${f}_{spat}$, ${f}_{year}$, and ${f}_{iy}$ are respectively functions suitable for space, year and space-year interaction. The spatial components ${f}_{spat}$ are disintegrated into two i.e., spatially unstructured ${f}_{unstr}$ and spatially structured ${f}_{str}$ effects. However, ${f}_{year}$ show the random year effects and this is modelled as a first-order random walk or AR(1) according to [29]. While ${f}_{iy}\left({s}_{i},y\right)$ is a space-year interaction.

To ascertain the wellness of the estimators, these seven models were compared as follows.

Model 1: $${\eta }_{iky}={\mathrm{\rm X}}_{iky}^{^{\prime}}\beta +{f}_{str}\left({s}_{i}\right)+{f}_{unstr}\left({s}_{i}\right)$$

Model 2: $${\eta }_{iky}={\mathrm{\rm X}}_{iky}^{^{\prime}}\beta +{f}_{str}\left({s}_{i}\right)+{f}_{unstr}\left({s}_{i}\right)+{\beta }_{y}$$

Model 3: $${\eta }_{iky}={\mathrm{\rm X}}_{iky}^{^{\prime}}\beta +{f}_{str}\left({s}_{i}\right)+{f}_{unstr}\left({s}_{i}\right)+{f}_{year} \left(y\right)$$

Model 4: $${\eta }_{iky}={\mathrm{\rm X}}_{iky}^{^{\prime}}\beta +{f}_{str}\left({s}_{i}\right)+{f}_{unstr}\left({s}_{i}\right)+{f}_{iy}\left({s}_{i},y\right)$$

Model 5: $${\eta }_{iky}={\mathrm{\rm X}}_{iky}^{^{\prime}}\beta +{f}_{str}\left({s}_{i}\right)+{f}_{unstr}\left({s}_{i}\right)+{\beta }_{y}+{f}_{iy}\left({s}_{i},y\right)$$

Model 6: $${\eta }_{iky}={\mathrm{\rm X}}_{iky}^{^{\prime}}\beta +{f}_{str}\left({s}_{i}\right)+{f}_{unstr}\left({s}_{i}\right)+{f}_{year} \left(y\right)+{f}_{iy}\left({s}_{i},y\right)$$

Model 7: $${\eta }_{iky}={\mathrm{\rm X}}_{iky}^{^{\prime}}\beta +{f}_{str}\left({s}_{i}\right)+{f}_{unstr}\left({s}_{i}\right)+{f}_{1year} \left(y\right)+{f}_{iy}\left({s}_{i},y\right)$$

were,

- ${\mathrm{\rm X}}_{iky}$ signifies the vector of categorical variables effects for child $k$ in state $i$ during year $y$
- $\beta$ is a vector of regression coefficients
- ${\beta }_{y}$ means the year-specific fixed effects
- ${f}_{unstr}\left({s}_{i}\right)$ and ${f}_{str}\left({s}_{i}\right)$ are the unstructured and structured random effects respectively
- ${f}_{year}$ and ${f}_{1year}$ show the smooth functions of the temporal random effects
- ${f}_{iy}\left({s}_{i},y\right)$ signifies the spatial-year interaction effect

Model 1 simply takes care of the spatially structured random effects, and this accounts for unobserved significant factors that change spatially transversely over the states and spatially unstructured random effects that caters for undetected variables inside the states. Hereafter, by assuming a categorical variable, it will have a linear effect on malaria and anaemia. The temporal effect is not assumed by this model. Model 2 follow the same pattern but in addition assumes a linear year trend taken by ${\beta }_{y}$. On the other hand, Model 3 contains separable space and year random effect which takes care of the linear effect of categorical variables. Also, Model 4 and Model 1 are parallel to each other but additionally, Model 4 takes care of space and year interaction which captures differences that is not shown by the main effects. Regarding Model 5, the assumption is made on the linear effects of the categorical variables, spatial random effects, linear year trend and space year-interaction. While Model 6 and Model 7 are alike but vary in prior assumptions of the temporal random year effects (${f}_{year}$, ${f}_{1year}$). Additionally, both models take on linear effects of categorical variables, spatial random effects of the location, space, and year interaction. In other words, all models take on linear effects of categorical variables through the term ${\mathrm{\rm X}}_{iky}^{^{\prime}}$.

### Prior specifications

Here, for the spatio-temporal logistic regression models, the full Bayesian approach was adopted. Diffuse priors were allocated to fixed effects and linear year trend, intrinsic conditional autoregressive (iCAR) was used to model the spatially structured random effects, while the independent and identically distributed (i.i.d) Gaussian prior was assigned spatially unstructured random effects. A first-order random walk was used to model the temporal year random effects ${f}_{1year}$. Nevertheless, it is interesting to note that varied prior specifications for the temporally changeable year effects ${f}_{year}$ were given in the models and penalized spline was given to the spatio-temporal logistic regression model. Also, independent penalized splines for the logistic independent first-order autoregressive model were adopted to model the spatial year-specific effects (interaction).

### Parameter estimation

In this research, we discussed the procedure of parameter estimation of the spatio-temporal logistic regression model of malaria and anaemia using the fully Bayesian approach. With due consideration, every unspecified parameter assumed random variables and was given adequate prior distributions. The posterior of the priors is given as:

5 $$p\left(\varphi ,\psi |y\right)\propto L\left(y|\varphi ,\psi \right)p\left(\varphi ,\psi \right)$$

where $L\left(y|\varphi ,\psi \right)$ is the likelihood of the penalized spline and $p\left(\varphi ,\psi \right)$ are the prior distributions of the spatio-temporal logistic regression model. The latent Gaussian field is expressed as $\varphi =\left\{\left\{\beta \right\},\left\{{\beta }_{y}\right\},\left\{{f}_{str}\left(.\right)\right\},\left\{{f}_{unstr}\left(.\right)\right\},\left\{{f}_{year}\left(.\right)\right\},\left\{{f}_{1year}\left(.\right)\right\},\left\{{f}_{iy}\left(.\right)\right\}\right\}$ while the equivalent hyperparameters are shown as $\psi =\left\{{\varrho }_{str},{\varrho }_{unstr},{\varrho }_{year},{\varrho }_{1year},{\varrho }_{iy}\right\}$. Conjugate gamma priors $Gamma\left(1, 0.00005\right)$ were allocated to all hyperparameters while R-integrated nested Laplace approximation (INLA) package was used to estimate the parameters.

## Results

Table 2 shows the model fit values for the spatio-temporal logistic regression models of malaria which comprises Deviance Information Criteria (DIC) and the effective number of parameters (DP). Model 7 was chosen as a better model because it gave the least DIC value of 10819.24. Therefore, the presentation of results and interpretations are based on Model 7 which includes both linear and nonlinear effects as well as the spatio-temporal effects.

**Table 2:** Summary of the model comparisons of malaria

	Model 1	Model 2	Model 3	Model 4	Model 5	Model 6	Model 7
DIC	11122.29	11110.03	11114.02	10821.05	10820.58	10821.11	10819.24
DP	52.31	53.40	54.27	86.24	86.14	86.19	85.06

Table 3 provides the adjusted posterior odds ratios estimates (AOR) and 95% confidence interval for the best fitting model mentioned above. Here, the results for significant covariates were discussed alone. Regarding child’s age in months, the results showed an increase in the odds of malaria for all ages. In the same vein, there was a significant increase in the odds of malaria among children whose anaemic status is positive and those who live in rural area. On the other hand, odds of malaria decrease significantly with respect to wealth index and mother’s educational level (Secondary and Higher). Furthermore, household with electricity had significantly lower odds of malaria compared to a household without electricity.

**Table 3:** Adjusted posterior odd ratios estimates (AOR) of malaria with 95% confidence interval

Variables	Malaria (Model7)	
	AOR	95% CI
Child's age in months (grouped) (ref = 1)		
2	1.452*	(1.235, 1.707)
3	1.979*	(1.692, 2.315)
4	2.399*	(2.051, 2.807)
5	2.787*	(2.372, 3.277)
6	3.221*	(2.742, 3.787)
Source of drinking water (ref = Tap/Other water)		
Well water	0.957	(0.863, 1.061)
Has electricity (ref = No)		
Yes	0.819*	(0.715, 0.939)
Main wall material (ref = Wood/Other)		
Cement/Bricks	0.943	(0.813, 1.094)
Main floor material (ref = Earth/Other)		
Concrete\Ceramics	0.919	(0.807, 1.047)
Main roof material (ref = Wood/Other)		
Zinc/Metal	1.004	(0.887, 1.137)
Anaemic status (ref = No)		
Yes	2.963*	(2.657, 3.306)
Wealth index (ref = Poor)		
Middle	0.758*	(0.641, 0.897)
Rich	0.468*	(0.365, 0.601)
Mother's educational level (ref = No education)		
Primary	0.915	(0.794, 1.056)
Secondary	0.748*	(0.642, 0.871)
Higher	0.442*	(0.339, 0.574)
Sex(ref = Male)		
Female	1.019	(0.930, 1.118)
Has radio(ref = No)		
Yes	0.983	(0.884, 1.093)
Type of place of residence (ref = Urban)		
Rural	1.637*	(1.436, 1.867)
Has television (ref = No)		
Yes	0.947	(0.814, 1.103)

$$*$$means significant

$CI$ means Confidence Interval

**Fig. 4:**
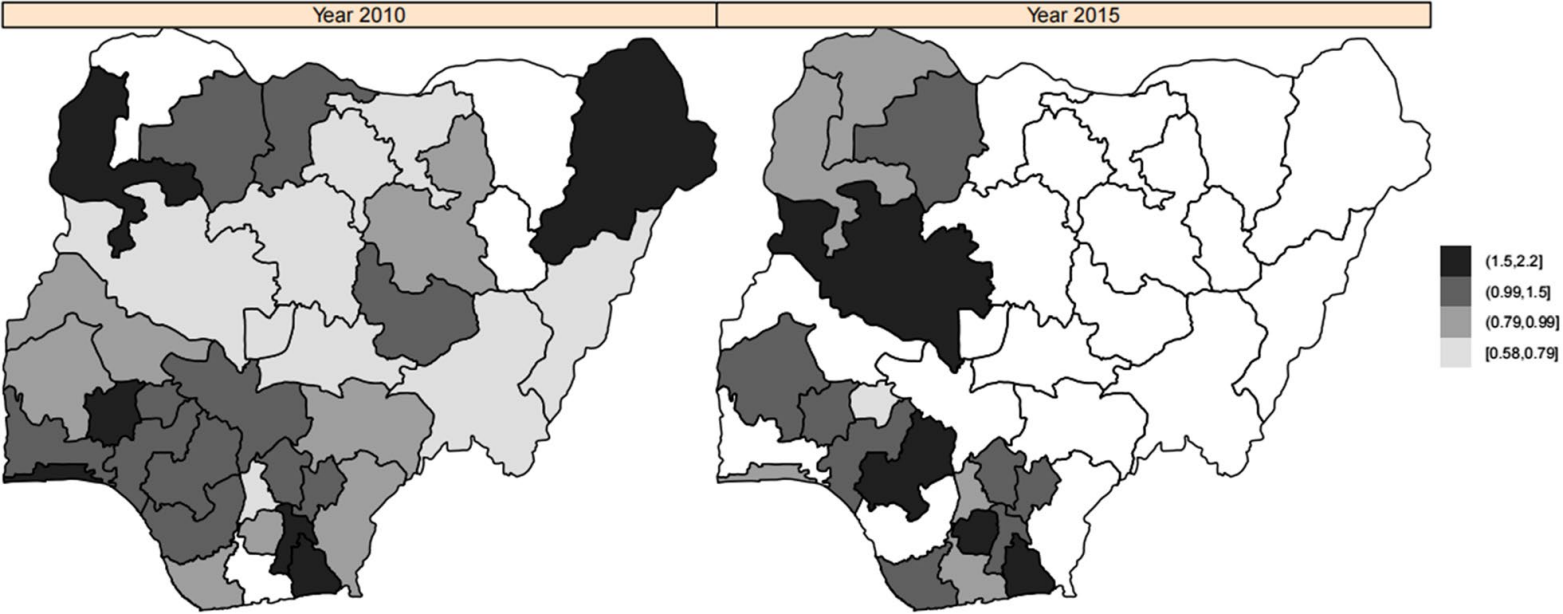
Maps displaying residual spatial effects of malaria in Nigeria for year 2010 and 2015 obtained from spatio-temporal interaction logistic regression model, i.e., Model 7

Figure 4 presents the mapped estimated residual spatial effects of the year 2010 and year 2015. The essence is to study how disease prevalence and risk factors change with time. These maps show unobserved spatial factors that are not captured in the survey or that capture the effects of cultural patterns. From the figure, there was an obvious spatial pattern change over the two years. Though higher concentrations in the two years are scattered, the states inside the Northeast, Northwest and Southwest regions have higher odds of 0.99–2.2 of malaria. In 2010, states in the north-east had higher odds of malaria but had lower odds of malaria later in 2015. .

Table 4 contains the results of $DIC$ and the effective number of parameters $DP$ of the model fit values for the spatio-temporal logistic regression models of anaemia.  From the summary, Model 6 gave the least DIC value of 10330.78. Therefore, the interpretation and presentation of results were based on this model.

**Table 4:** Summary of the model comparisons of anaemia

	Model 1	Model 2	Model 3	Model 4	Model 5	Model 6	Model 7
DIC	10472.33	10474.30	10472.42	10330.80	10330.85	10330.78	10330.87
DP	53.11	54.10	53.09	83.12	83.59	83.11	83.18

**Fig. 5:**
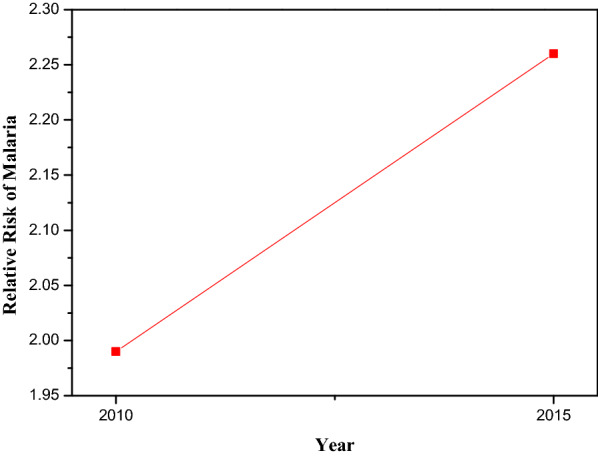
Depicting estimated posterior relative risk of malaria for the logistic regression best fitting model

Figure 5 shows the posterior relative risk of malaria. Here, there was an obvious change in the relative risk of malaria over the two years. This implies that there has been an increase in the relative risk of malaria from 2010 to 2015.

Table 5 presents the adjusted posterior odds ratios estimates (AOR) and 95% confidence interval for the best fitting model mentioned above. Discussion on the results was basically on the significant covariates. There was a significant decrease in odds of anaemia for children in age categories 3, 4, 5, and 6 but insignificant for  age category 2. Also, the odds of anaemia decrease significantly with increasing mother’s educational level. Furthermore, sex, household that has radio and television had significantly lower odds of anaemia. On the other hand, child’s age in months, the results showed that there was an increase in the odds of malaria but only significant for children in age categories 2, 3 and 6. While the odds of anaemia increased significantly for malaria rapid test results, source of drinking water, wealth index and type of place of residence.

**Table 5:** Adjusted posterior odd ratios estimates (AOR) of anaemia with 95% confidence interval

Variables	Malaria (Model7)	
	AOR	95% CI
Child's age in months (grouped) (ref = 1)		
2	0.910	(0.763, 1.086)
3	0.599*	(0.506, 0.708)
4	0.402*	(0.341, 0.473)
5	0.330*	(0.280, 0.390)
6	0.303*	(0.256, 0.358)
Source of drinking water (ref = Tap/Other water)		
Well water	1.134*	(1.021, 1.259)
Has electricity (ref = No)		
Yes	1.019	(0.881, 1.180)
Main wall material (ref = Wood/Other)		
Cement/Bricks	0.908	(0.774, 1.065)
Main floor material (ref = Earth/Other)		
Concrete\Ceramics	1.027	(0.900, 1.170)
Main roof material (ref = Wood/Other)		
Zinc/Metal	0.932	(0.818, 1.062)
Result of malaria rapid test (ref = No)		
Yes	2.985*	(2.676, 3.331)
Wealth index (ref = Poor)		
Middle	1.206*	(1.005, 1.448)
Rich	1.229	(0.946, 1.595)
Mother's educational level (ref = No education)		
Primary	0.849*	(0.730, 0.987)
Secondary	0.795*	(0.680, 0.928)
Higher	0.659*	(0.526, 0.825)
Sex (ref = Male)		
Female	0.784*	(0.713, 0.862)
Has radio (ref = No)		
Yes	0.778*	(0.695, 0.871)
Type of place of residence (ref = Urban)		
Rural	1.475*	(1.295, 1.679)
Has television (ref = No)		
Yes	0.789*	(0.673, 0.925)

$$*$$means significant

$CI$ means Confidence Interval

**Fig. 6:**
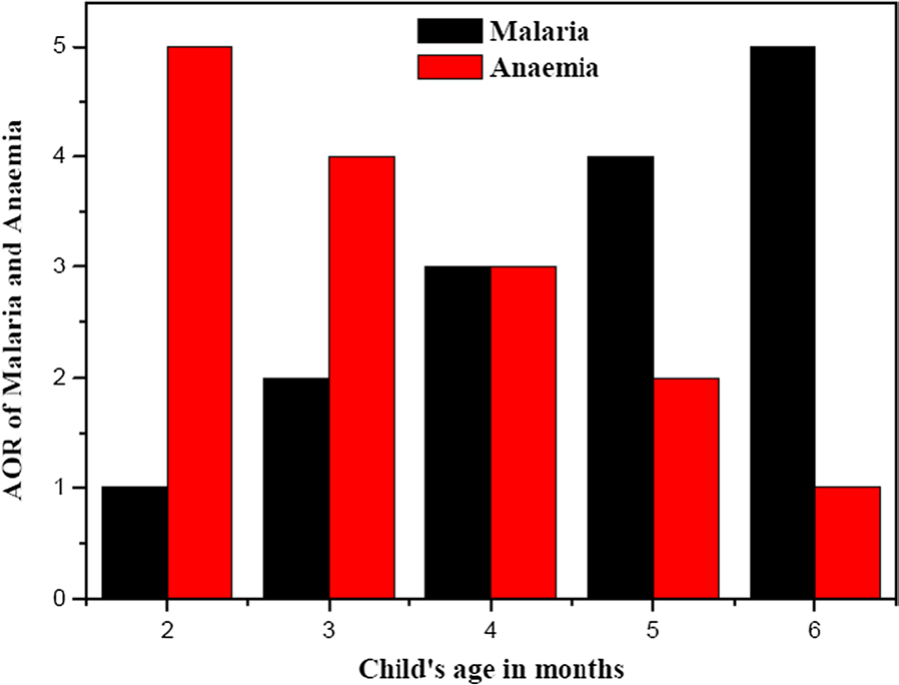
Relationship between child’s age in months and AOR of malaria and anaemia

Figure 6 is the graphical representation of child’s age in months and the adjusted odd ratio (AOR) of malaria and anaemia. The relationship between child’s age in months and the adjusted odd ratio of malaria increase significantly across the age group. While anaemia decreases significantly. This might be due to the stimulation of antimalarial immune defenses by malaria antigen in breast milk which reduces malaria risk in infants attributable to breastfeeding [30]. As children are weaned, they are vulnerable to malaria as they have lost maternal immunity and are yet to develop self-immunity against infection [31]. As a result, there will be a decrease in anaemia infection because individuals at some point develop a disease-controlling immunity that makes them asymptomatic [32].

**Fig. 7:**
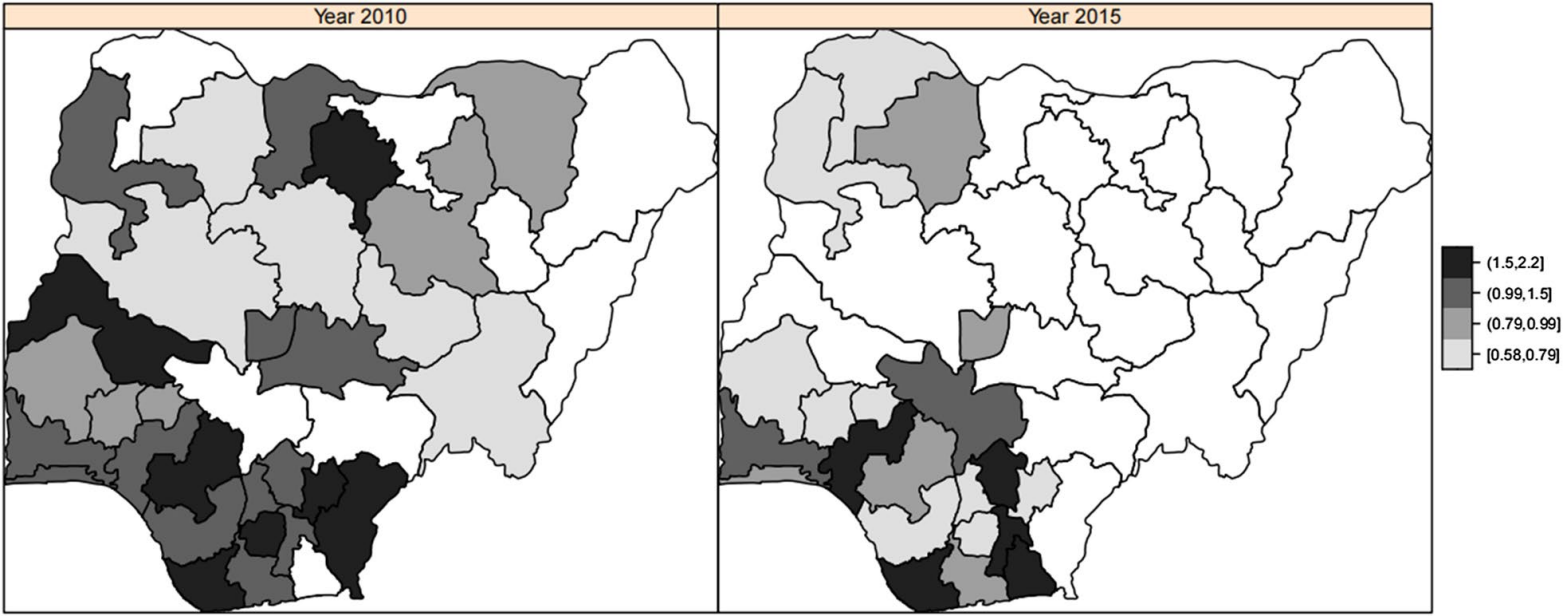
Maps displaying residual spatial effects of anaemia in Nigeria for year 2010 and 2015 sprang from the spatio-temporal interaction logistic regression model i.e., Model 6

Figure 7 displays the mapped estimated adjusted posterior odds of residual spatial effects of the year 2010 and 2015 in Nigeria. In our first work, we focused on the evolution of the geographical variation of anaemia. The map in Fig. 4 represents the estimated residual spatial effects for the two years. The colours for the regions are the same as described above. In both years, the South-South and partially South-West regions showed a higher concentration of anaemia with odds ratio of 0.90–2.2. Notwithstanding, in 2010, some part of North-Central region had a higher concentration of the same odds ratio. Other things seem similar to the previous figure.

**Fig. 8:**
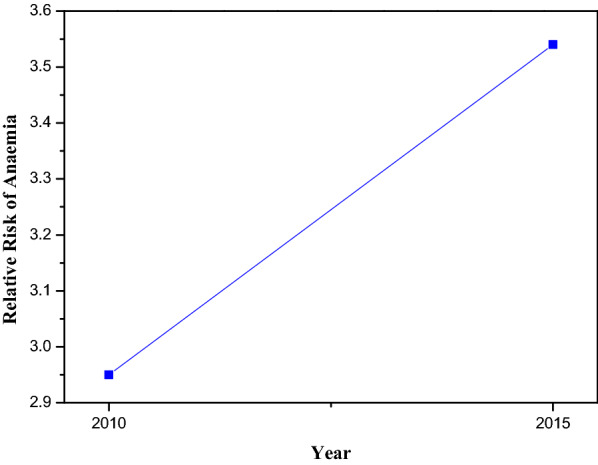
Depicting estimated posterior relative risk of anaemia for the logistic regression best fitting model

Figure 8 provides the estimated posterior relative risk of anaemia. There was an increase in the relative risk of anaemia in the two years i.e., the transmission of anaemia infection was high in 2015.

## Discussion

This research work applied spatio-temporal models to investigate the relative risks and geographical variation of malaria and anaemia in Nigeria. This research work was carried out with the sole aim of developing and applying the exact statistical models to assess determinant factors and geographical distinctions of malaria and anaemia. In addition, to apply a unified framework of flexible models within Bayesian hierarchical modelling to understand various factors associated with this discreet type of malaria and anaemia prevalence among children from 0 to 59 months in Nigeria. The models considered are an augmentation to classical models which include spatial and spatio-temporal models for identification of geographical variation of year-specific effects. Logistic regression was developed to assess influential factors and state variation of malaria and anaemia prevalence. The structured additive modelling approach gives allowance for different kinds of predictors to be included in classical models in an additive manner by borrowing strength from both parametric and non-parametric models. Integrated nested Laplace approximation was used to investigate the spatio-temporal effect on childhood malaria and anaemia disease with the application of MIS (Malaria indicator survey) datasets in Nigeria. For each model, the Deviance Information Criteria (DIC) were compared, and the best model was used to fit malaria and anaemia data of Nigeria. Among the models considered, the spatio-temporal interaction logistic regression model was chosen as the best model to fit malaria data while for anaemia, model 6 (interaction with one random time effect (autoregressive prior of order 1(AR1))) was chosen. For both diseases, variation can be seen among the Nigeria states and clustering among states with high malaria and anaemia relative risk (RR). Child’s age in month, main wall material, anaemic status, wealth index, mother’s educational level and type of place of residence were significantly related to malaria and anaemia except for the source of drinking water, sex, and household that has radio and television that were significantly related to only anaemia over the two years period, this is in line with [33, 34]. While anaemia is seen as a dominant determinant of malaria, malaria as well as a major determinant of anaemia[35, 36]. In the two years considered, we found out that the states within the northern, southern, and western regions have the higher prevalence of malaria and anaemia. The lowest prevalence of malaria and anaemia was seen in states within the eastern region. Also, we estimated year temporal effects on malaria and anaemia. For both diseases, the plots showed no obvious change in the spread of malaria and anaemia.

There are always limitations in every research. In this study, the main limitation is the number of years available for us to estimate the spatio-temporal trends of malaria or anaemia. This matter poses a hinderance to investigating the trend of malaria or anaemia pandemic during the early years. Also, this study used secondary data from cross-sectional surveys which did not allow the causal relationships to be established. Furthermore, though iron deficiency is one of the major causes of anaemia, there was no information on iron levels in children. Notwithstanding the limitations, the strength of this study is in the use of individual-level malaria RDT results instead of indicators or estimates of malaria or anaemia.

## Conclusion

There is room for further investigation on this research work. In this work, FB approach within the Bayesian hierarchical modelling to model malaria and anaemia prevalence in Nigeria. Furthermore, the Bayesian structured additive approach was used to model the determinants of malaria and anaemia. The findings from this work show that there will be likely a reduction in the spread of these diseases if commendations are adequately adhered to. Therefore, government should focus on improving mother’s education and standard of living. Also, pertaining to these diseases, there should be aggressive awareness on social television programs.

## Data Availability

Data is available in Demographic Health Survey. Public access to databases is closed (requires a password) (http://www.dhsprogram.com/data/dataset_admin/login_main.cfm). We received permission from DHS to use the data via a password supplied by DHS Admin. (Authorization letter is attached as supplementary material).
